# Dietary supplementation with *Lactobacillus plantarum* and *Bacillus velezensis* improves growth performance, antioxidant capacity, immunity and intestinal microbiota in growing-finishing pigs

**DOI:** 10.3389/fmicb.2026.1913467

**Published:** 2026-09-17

**Authors:** Zijing Zhang, Zihe Li, Yingmei Zhang, Xinyu Wu, Xiaojia Wu, Xi Chen, Liang Peng, Qinghua Liu, Changchuan Ye

**Affiliations:** 1 Engineering Research Center for Animal Breeding and Sustainable Production, College of Animal Science, Fujian Agriculture and Forestry University, Fuzhou, China; 2 Fujian Academy of Agricultural Sciences, Fuzhou, China; 3 Fujian Huatian Ecological Agriculture and Animal Husbandry co., Ltd, Fuzhou, China

**Keywords:** antioxidant, *Bacillus velezensis*, growing pigs, immunity, intestinal microbiota, *Lactobacillus plantarum*, probiotics

## Abstract

**Introduction:**

Compound probiotics have gained increasing recognition for their ability to improve growth performance and animal health in animal production. This study evaluated the effects of dietary supplementation with *Lactobacillus plantarum* QZF (10^11^ CFU/d), *Bacillus velezensis* BD01 (10^12^ CFU/d) or their combination on growth performance, serum biochemistry, antioxidant capacity, immunity and gut microbiota in growing pigs.

**Methods:**

A total of 160 growing Landrace × Yorkshire gilts were randomly assigned to four treatment groups: CON (basal diet), SPL (basal diet supplemented with *L. plantarum* fermentation broth), SPB (basal diet supplemented with *B. velezensis* fermentation broth), and DP (basal diet supplemented with both strains fermentation broth). Each group consisted of 40 pigs (8 replicates of 5 pigs), and the experiment lasted 47 days.

**Results:**

The SPB and DP groups showed significantly higher average daily gain and serum total protein than CON group, attributed to the main effect of *B. velezensis* (*p*_(B)_ < 0.01). The SPL, SPB, and DP groups showed significant increases in serum total cholesterol, free fatty acids and urea nitrogen (*p*_(L)_ < 0.01 for TC and FFA; *p*_(L)_ < 0.01, *p*_(B)_ < 0.01, and *p*_(L × *B*)_ < 0.01 for BUN). Serum antioxidant enzyme activities (SOD, CAT, and GSH-Px) were significantly affected by both the main effect of *L. plantarum* and the main effect of *B. velezensis*, with no significant L × B interaction. In contrast, total antioxidant capacity (T-AOC) was significantly affected only by the main effect of *B. velezensis*, whereas neither the main effect of *L. plantarum* or the L × B interaction was significant. For immune parameters, ALB and IgG were increased by *B. velezensis* (*p*_(B)_ < 0.05), while IgM and IgA were elevated by *L. plantarum* (*p*_(L)_ < 0.05). No interaction was detected for these parameters. Microbiota analysis revealed increased alpha diversity (ACE and Chao1) and altered microbial composition, with *B. velezensis* increasing *Proteobacteria* and the compound probiotic enriching *Actinobacteriota* and *Lactobacillales*.

**Discussion:**

In conclusion, dietary probiotics, especially the multi strain combination, positively influenced growth performance, immune function, and antioxidant capacity in growing pigs, representing a promising nutritional strategy for swine production.

## Introduction

The use of sub-therapeutic doses of antibiotics in animal feed is a common practice in livestock production, which employed to control infectious diseases and enhance growth performance (Cheng et al., 2014). However, the global rise in antibiotic resistance has become a growing concern in the field of animal health (Mevius and Hellebrekers, 2010). Since 2006, the European Union has completely banned the addition of antibiotics to animal feed. In China, a complete ban on antibiotics in animal feed has also been implemented in 2020. The overuse and misuse of antibiotics have exacerbated resistance issues, highlighting the urgent need to develop safe and effective alternatives for sustainable livestock production (Singer et al., 2019; Zamojska et al., 2021).

In pig production, intestinal health is a key determinant of growth efficiency, nutrient utilization, and disease resistance. Various stressors, including dietary transitions, environmental changes, and pathogenic challenges, can disturb intestinal microbial homeostasis, resulting in impaired nutrient absorption, depressed growth performance and increased incidence of intestinal disorders (Rymut et al., 2021; Tang et al., 2022; Gormley et al., 2024). These issues are especially prominent during the growing period, when pigs undergo rapid development and experience intensive metabolic demands. Dietary probiotics have been recognized as an effective nutritional strategy to alleviate these problems and improve intestinal health and overall production performance.

Probiotics are regarded as promising alternatives to antibiotics due to their beneficial effects on host health and pathogen inhibition. Beneficial microorganisms, such as *Lactobacillus, Bacillus* and *Saccharomyces cerevisiae* have been widely applied in livestock, especially in swine production (Kenny et al., 2011). As viable microbial supplements, probiotics can modulate gut microbial composition, enhance intestinal barrier function and strengthen host immune defense, thereby reducing the risk of intestinal diseases (Chandrasekaran et al., 2024). Previous studies have demonstrated that probiotics can alleviate intestinal inflammation by suppressing the expression of pro-inflammatory cytokines and inducing specific regulatory responses in the host (Borchers et al., 2009).

*Lactobacillus plantarum* is a widely used lactic acid bacterium with strong environmental adaptability and high genomic plasticity. It can produce a variety of bacteriocins with potent antimicrobial activity, showing great potential in food preservation and as an adjunct to antibiotics (Seddik et al., 2017). Dietary supplementation with *Lactobacillus species* effectively improves growth performance, maintains gastrointestinal microbial balance, and reduces pathogen infection. Studies have confirmed that *L. plantarum* reduces diarrhea rate and improves intestinal health in pigs (Khongkool et al., 2025; Yang et al., 2022). *Bacillus velezensis* is another promising probiotic in the livestock industry (Brutscher et al., 2024). Research has shown that *B. velezensis* promotes growth, enhances immune function and metabolic status, and improves intestinal health and microbial composition in livestock and poultry (Yu et al., 2025; Zhu La et al., 2024). Recently, multi-strain probiotic preparations have attracted increasing attention. Compared with single-strain probiotics, compound probiotics often exert synergistic effects and provide more comprehensive benefits, mainly by inhibiting pathogenic colonization and maintaining intestinal microbial balance (Kim et al., 2024; Li et al., 2023).

Collectively, probiotic supplementation effectively improves growth, immunity and intestinal health in pigs, and multi-strain combinations often show superior effects. This study was conducted to evaluate the effects of dietary supplementation with *Lactobacillus plantarum* QZF, *Bacillus velezensis* BD01 or combination of them on growth performance and intestinal health in growing pigs. The results will provide a theoretical basis and practical reference for the application of multi-strain probiotics as antibiotic alternatives in the swine feed industry.

## Materials and methods

### Experimental design and feeding management

A total of 160 piglets were divided into four treatment groups. The piglets were randomly allocated to the four groups without stratification based on initial body weight. Despite the random allocation process, significant differences in initial body weight still existed across treatment groups. For this reason, ANCOVA was used in subsequent analyses, yet this method could only partially mitigate the above confounding effect. All animals were randomly allocated to four experimental groups, namely CON group, SPL group, SPB group and DP group, with 40 pigs in each group. The 40 pigs within each treatment group were housed in 8 separate pens, containing 5 pigs per pen. The basal diet was formulated to meet the nutritional requirements of growing pigs, and its composition is detailed in Table 1.

**Table 1:** Ingredient composition and Nutrient composition of the basal diet (air-dried basis).

Item	Contents
**Ingredient composition (%)**	
Corn	68.00
Soy bean meal, 43%	23.00
Wheat bran	5.00
Premix1	4.00
**Nutrition content 2 (%)**	
DE (MJ/kg)	13.78
NE (MJ/kg)	10.60
Crude protein (%)	14.27
Ether extract (%)	3.61
Ash (%)	5.27
Acid detergent fiber (%)	7.79
Neutral detergent fiber (%)	17.18
Dry matter (%)	87.96
Calcium (%)	0.88
Phosphorus (%)	0.54

1 Premix provided the following per kilogram of diet: vitamin A, 5500.00 IU; vitamin D, 2200.00 IU; vitamin E, 30.00 IU; vitamin K3, 2.50mg; vitamin B1, 1.50 mg; vitamin B2, 3.00 mg; vitamin B6, 3.00 mg; vitamin B12, 27.60 μg; nicotinic acid, 30.00 mg; pantothenic acid, 13.80 mg; biotin, 44.00mg; folacin, 0.70 mg; choline, 400.00 mg; Mn, 40.00 mg; Fe, 75.00 mg; Zn, 75.00 mg; Cu, 100.00 mg; I, 0.30 mg; Se, 0.30 mg.

2 DE and NE were calculated value. Crude protein (CP), ether extract (EE), crude ash (Ash), acid detergent fiber (ADF), Neutral detergent fiber (NDF), dry matter (DM), calcium (Ca) and phosphorus (P) were measured and recorded. CP was determined by the Kjeldahl method; EE was determined using the Soxhlet extraction method; ADF and NDF were measured using the filter bag technique; DM was determined using the oven-drying method; Ca was determined by the EDTA complexometric titration method; P was measured by spectrophotometry at a wavelength of 400 nm with the molybdenum blue reaction.

As illustrated in Figure 1, two probiotic strains were used in this study: *Lactobacillus plantarum* QZF (CGMCC No. 31,389) and *Bacillus velezensis* BD01 (CGMCC No. 37,239). The probiotic powders, which contained commercially formulated inorganic mineral elements, were provided by Huiying Animal Health Co., Ltd. (Xiamen, China) and stored in sealed, moisture-proof bags at 4 °C until use.

**Figure 1:**
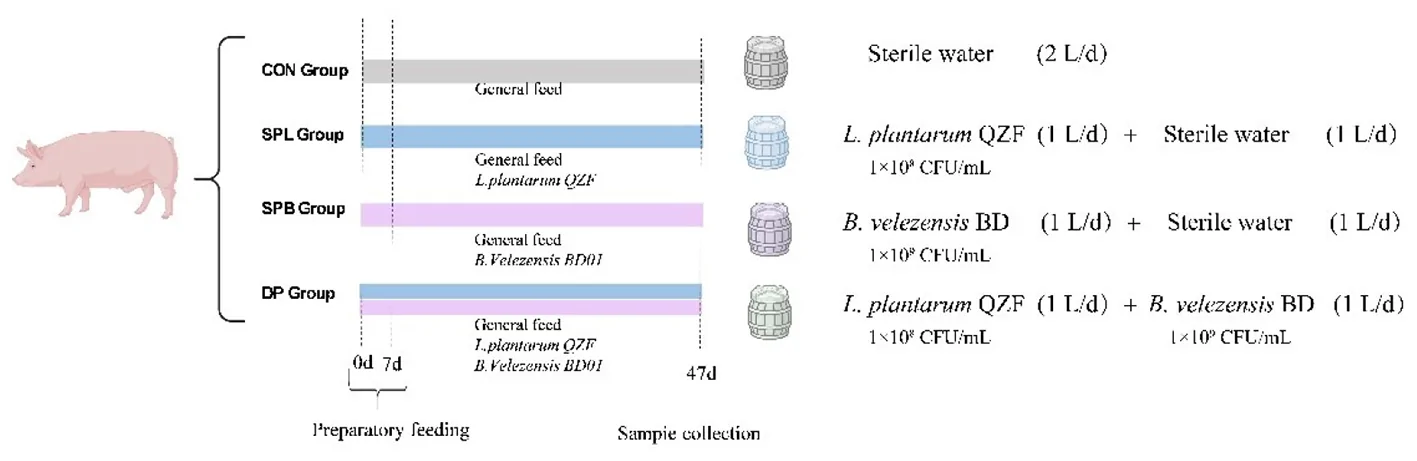
The schematic diagram of experimental design in this study.

For preparation, 50 L of water, 750 g of brown sugar, and 500 g of probiotic powder were accurately weighed and thoroughly mixed. The mixture was fermented under anaerobic conditions at 30 °C for 24 h. After fermentation, the viable count of *L. plantarum* in the fermented suspension was approximately 1 × 10^8^ CFU/mL, and the viable count of *B. velezensis* was approximately 1 × 10^9^ CFU/mL. The fermented suspensions were freshly prepared and supplied to the corresponding experimental groups every morning between 8:00 and 9:00.

In addition to *ad libitum* drinking water, the CON group received 2 L of sterile water per pig daily as a supplement. The SPL group received 1 L of *L. plantarum* suspension (1 × 10^8^ CFU/mL) and 1 L of sterile water per pig daily. The SPB group received 1 L of *B. velezensis* suspension (1 × 10^9^ CFU/mL) and 1 L of sterile water per pig daily. The DP group received 1 L of *L. plantarum* suspension (1 × 10^8^ CFU/mL) and 1 L of *B. velezensis* suspension (1 × 10^9^ CFU/mL) per pig daily. All suspensions were provided as drinking water in separate containers to ensure complete consumption. The daily administration volume of 1 L per pig corresponds to a total daily intake of approximately 1 × 10^11^ CFU for *L. plantarum* and 1 × 10^12^ CFU for *B. velezensis*.

Before the start of the experiment, all pens were thoroughly cleaned and disinfected. Pigs were assigned to their respective groups and individually identified. Routine disease prevention measures were implemented according to standard protocols. Throughout the trial, the pig house was maintained under natural lighting and ventilation. All pigs had *ad libitum* access to feed and water. Ambient temperature and humidity were maintained within normal ranges, and pens were disinfected once weekly. Immunizations were administered in accordance with the routine management practices. Animals were monitored daily for general health status, behavioral responses, feed intake, constipation, diarrhea and mortality.

### Growth performance

On days 0 and 47 of the experiment, all pigs were individually weighed in the morning after a 12-h overnight fast. The pen (*n* = 8 per treatment group, with 5 pigs per pen) was considered the experimental unit for growth performance analysis. Initial body weight (IBW), final body weight (FBW), and average daily gain (ADG) were calculated for each individual pig, and the pen-mean values were then used for statistical analysis. Fecal consistency was monitored at fixed times each week throughout the experimental period, and the diarrhea rate was calculated for each pen based on the recorded observations, with pen-level data used for subsequent statistical comparison.

### Blood sampling and biochemical analysis

On day 47 of the experiment, after a 12-h overnight feed withdrawal, blood samples were collected from the anterior vena cava using serum separator vacuum tubes. In each treatment group, one pig was randomly selected from each of the eight pens, yielding eight serum samples per group. From these eight samples, six were randomly selected for subsequent analyses. The collected blood samples were centrifuged at 4,000 rpm for 10 min at 4 °C. The supernatant serum was transferred into sterile centrifuge tubes and stored at −20 °C prior to the determination of biochemical, antioxidant, and immune parameters. Repeated freeze–thaw cycles were strictly avoided during storage and analysis.

### Fecal sampling and microbial analysis

Fresh fecal samples were collected on day 0 and day 47 of the trial after a 12-h overnight feed withdrawal. In each treatment group, one pig was randomly selected from each of the eight pens, yielding eight fecal samples per group. From these eight samples, six were randomly selected for subsequent analyses. All samples were freshly collected from the pen floor immediately after defecation and transferred into sterile 50 mL centrifuge tubes. All tubes were stored at −20 °C until analysis of gut microbial community composition and calprotectin concentrations.

### Serum biochemical parameters, antioxidant capacity and immunity

Serum biochemical parameters were determined using an automated biochemical analysis system (Fuzhou Melisa Biotechnology Co., Ltd., Fuzhou, China). Concentrations of glucose, triglycerides, high-density lipoprotein cholesterol, low-density lipoprotein cholesterol, total protein, total cholesterol, and albumin were measured using commercial ELISA kits (Hunan Yonghe Sunshine Technology Co., Ltd., Hunan, China) following the manufacturer's instructions. Serum free fatty acids and urea nitrogen were determined using commercial assay kits (Nanjing Jiancheng Bioengineering Institute, Nanjing, China) according to the protocol.

For antioxidant capacity, the activities of superoxide dismutase, catalase, and glutathione peroxidase, as well as malondialdehyde content and total antioxidant capacity, were measured using corresponding commercial assay kits (Shanghai Liquid Mass Testing Technology Co., Ltd., Shanghai, China).

For immune assessment, serum concentrations of immunoglobulin A, immunoglobulin G, and immunoglobulin M were determined using commercial ELISA kits (Shanghai Yimei Biotechnology Co., Ltd., Shanghai, China).

### Fecal calprotectin and microbial composition analysis

Fecal calprotectin levels were determined using a commercial ELISA kit (Shanghai Yimei Biotechnology Co., Ltd., Shanghai, China) according to the manufacturer's protocol. Measurements were performed on fecal samples collected at both the start (day 0) and the end (day 47) of the trial, and the change in calprotectin content (end value minus start value) was calculated for each pig.

To evaluate the effects of dietary probiotic supplementation on the intestinal microbiota of growing pigs, 16S rRNA high-throughput sequencing was performed (Shanghai Yapu Biotechnology Co., Ltd., Shanghai, China). Total microbial DNA was extracted from fecal samples collected at the end of the trial using the cetyltrimethylammonium bromide method following the manufacturer's instructions. The V3–V4 region of the bacterial 16S rRNA gene was amplified with universal primers 338F (ACTCCTACGGGAGGCAGCA) and 806R (GGACTACHVGGGTWTCTAAT), with sequencing adapters added to the primer ends. The amplified products were purified, quantified, normalized, and used to construct a sequencing library. After quality inspection, the library was sequenced on the Illumina NovaSeq 6,000 platform.

Raw sequencing reads were quality-filtered using Trimmomatic v0.33. Primer sequences were removed using cutadapt 1.9.1 to obtain clean reads. Denoising, including paired-end merging and chimera removal, was performed with the DADA2 algorithm in QIIME2 2020.6, generating high-quality amplicon sequence variants (ASVs). Taxonomic annotation of ASVs was conducted using the Silva 138 database and a naive Bayes classifier, providing taxonomic information from phylum to species level. A species abundance table was generated using QIIME software, and community structure visualization was performed with R software based on the ASV abundance table.

### Statistics analysis

Statistical analyses were performed using IBM SPSS Statistics version 27.0 (IBM Corp., Armonk, NY, USA). Data analysis was performed using two-way analysis of variance (ANOVA) or two-way analysis of covariance (ANCOVA). For growth performance (final body weight and average daily gain), two-way ANCOVA was adopted with the initial body weight set as the covariate. For the other measured indices, two-way ANOVA was used for statistical analysis. The main effects of *L. plantarum* supplementation (L)*, B. velezensis* supplementation (B) and their interaction (L × B) were first evaluated. Pairwise comparisons among treatment groups were performed using Tukey's multiple comparison test only when a significant interaction was detected. Results are presented as mean ± standard error of the mean (SEM). In this study, statistical significance was set at *p* < 0.05.

## Results

### Growth performance

As shown in Table 2, initial body weight differed significantly among groups [*p*_(L)_ = 0.37, *p*_(B)_ = 0.13, *p*_(L × *B*)_ < 0.01] using two-way ANOVA. In this case, final body weight (FBW) and average daily gain (ADG) were analyzed using two-way analysis of covariance (ANCOVA) with initial body weight as a covariate. Other parameters were analyzed by two-way ANOVA. Dietary supplementation with *B. velezensis* (B) significantly increased FBW and ADG [*p*_(B)_ < 0.01], whereas *L. plantarum* (L) alone had no significant effect on either parameter [*p*_(L)_ = 0.76 and 0.60, respectively]. A significant L × B interaction was detected only for ADG [*p*_(L × *B*)_ = 0.01]. Although the DP group showed a higher ADG than the CON and SPL groups, its mean ADG was slightly lower than that of the SPB group. Supplementation of *L. plantarum* significantly reduced diarrhea rate [*p*_(L)_ < 0.01], while *B. velezensis* had a trend toward reducing diarrhea rate [*p*_(B)_ = 0.05]. No interaction was observed for diarrhea rate [*p*_(L × *B*)_ = 0.38].

**Table 2:** Effects of dietary probiotic supplementation on growth performance.

Items	CON(L-B-)	SPL(L+B-)	SPB(L-B+)	DP(L+B+)	*p*-value		
					L	B	L×B
FBW (kg)	65.41 ± 0.80	67.10 ± 1.17	69.64 ± 1.02	68.61 ± 0.70	0.73	< 0.01	0.16
ADG (g)	853.13 ± 13.42a	889.50 ± 12.02ab	944.10 ± 7.60c	922.88 ± 9.95bc	0.50	< 0.01	0.01
Diarrhea rate (%)	8.40 ± 0.20	7.90 ± 0.14	8.03 ± 0.09	7.76 ± 0.04	< 0.01	0.04	0.38

FBW, final body weight; ADG, average daily gain. For FBW and ADG, the results are presented as the adjusted mean ± SEM. For diarrhea rate, the results are presented as the mean ± SEM. The original mean of initial body weight (IBW) and FBW were shown in Table S1. *n* = 8. When a significant interaction was observed, different letters indicate significant differences based on Tukey's multiple comparisons test (*P* < 0.05).

### Serum biochemical parameters

As shown in Table 3, no significant L × B interaction was observed for total protein (TP) and albumin (ALB) [*p*_(L × *B*)_ = 0.94 and 0.21, respectively]. Dietary supplementation with *B. velezensis* (B) significantly increased the contents of total protein (TP) and albumin (ALB) [*p*_(B)_ < 0.01], while single treatment with *L. plantarum* had no significant effect on TP and ALB [*p*_(L)_ = 0.32 and 0.21, respectively]. Dietary supplementation with *L. plantarum* significantly increased total cholesterol (TC) and free fatty acids (FFA) [*p*_(L)_ < 0.01], while single supplementation with *B. velezensis* had no significant effect on TC and FFA [*p*(B) = 0.38 and 0.15, respectively]. Meanwhile, both *L. plantarum* and *B. velezensis* significantly increased blood urea nitrogen (BUN) [*p*_(L)_ < 0.01, *p*_(B)_ < 0.01], and a significant interaction was observed [*p*_(L × *B*)_ < 0.01]. Nevertheless, both *Lactobacillus plantarum* and *Bacillus velezensis* exerted significant main effects, and a significant interaction between the two strains was observed for BUN (*p* < 0.01). The BUN concentration in the DP group was lower than that in both single-strain groups, suggesting a possible antagonistic effect between the two strains in BUN.

**Table 3:** Effects of dietary probiotic supplementation on serum biochemical parameters.

Item	CON(L-B-)	SPL(L+B-)	SPB(L-B+)	DP(L+B+)	*p*-value		
					L	B	L×B
Glu (mmol/L)	3.80 ± 0.19	3.52 ± 0.15	3.87 ± 0.23	3.95 ± 0.19	0.61	0.22	0.37
TG (mmol/L)	0.45 ± 0.06	0.37 ± 0.06	0.46 ± 0.06	0.36 ± 0.09	0.21	0.99	0.89
HDL-C (mmol/L)	0.68 ± 0.04	0.76 ± 0.08	0.83 ± 0.03	0.80 ± 0.03	0.62	0.08	0.29
LDL-C (mmol/L)	0.59 ± 0.03	0.62 ± 0.06	0.62 ± 0.04	0.57 ± 0.03	0.82	0.82	0.36
TP (g/L)	49.95 ± 1.10	51.38 ± 1.97	55.30 ± 1.10	56.98 ± 1.68	0.32	< 0.01	0.94
TC (mmol/L)	2.45 ± 0.15c	4.77 ± 0.15a	3.48 ± 0.12b	3.47 ± 0.17b	< 0.01	0.38	< 0.01
FFA (mmol/L)	0.38 ± 0.04c	0.95 ± 0.04a	0.75 ± 0.02b	0.69 ± 0.04b	< 0.01	0.15	< 0.01
BUN (mmol/L)	4.03 ± 0.38b	7.71 ± 0.26a	7.22 ± 0.19a	6.71 ± 0.44a	< 0.01	< 0.01	< 0.01
ALB (g/L)	30.58 ± 0.78	30.57 ± 1.68	32.72 ± 1.11	35.85 ± 0.99	0.21	< 0.01	0.21

Glu, Glucose; TG, Triglyceride; HDL-C, High-density lipoprotein; LDL-C, Low-density lipoprotein; TP, Total protein; TC, Total cholesterol; FFA, Free Fatty Acids; BUN, Urea nitrogen; ALB, Albumin. The results are presented as the mean ± SEM. *n* = 6. When a significant interaction was observed, different letters indicate significant differences based on Tukey's multiple comparisons test (*p* < 0.05).

### Serum antioxidant capacity

As shown in Figure 2, no significant L × B interaction was observed for superoxide dismutase (SOD), catalase (CAT), or glutathione peroxidase (GSH-Px) [*p*_(L × *B*)_ = 0.79, 0.67, and 0.73, respectively]. Similarly, no interactive effect was detected for total antioxidant capacity (T-AOC) [*p*_(L × *B*)_ = 0.94]. Dietary supplementation with *B. velezensis* significantly increased T-AOC activity [*p*_(B)_ = 0.02], whereas *L. plantarum* alone had no significant effect on T-AOC [*p*_(L)_ = 0.55]. Both *L. plantarum* and *B. velezensis* markedly elevated the levels of SOD, CAT, and GSH-Px [*p*_(L)_ < 0.01 for all three; *p*_(B)_ < 0.05 for all three].

**Figure 2:**
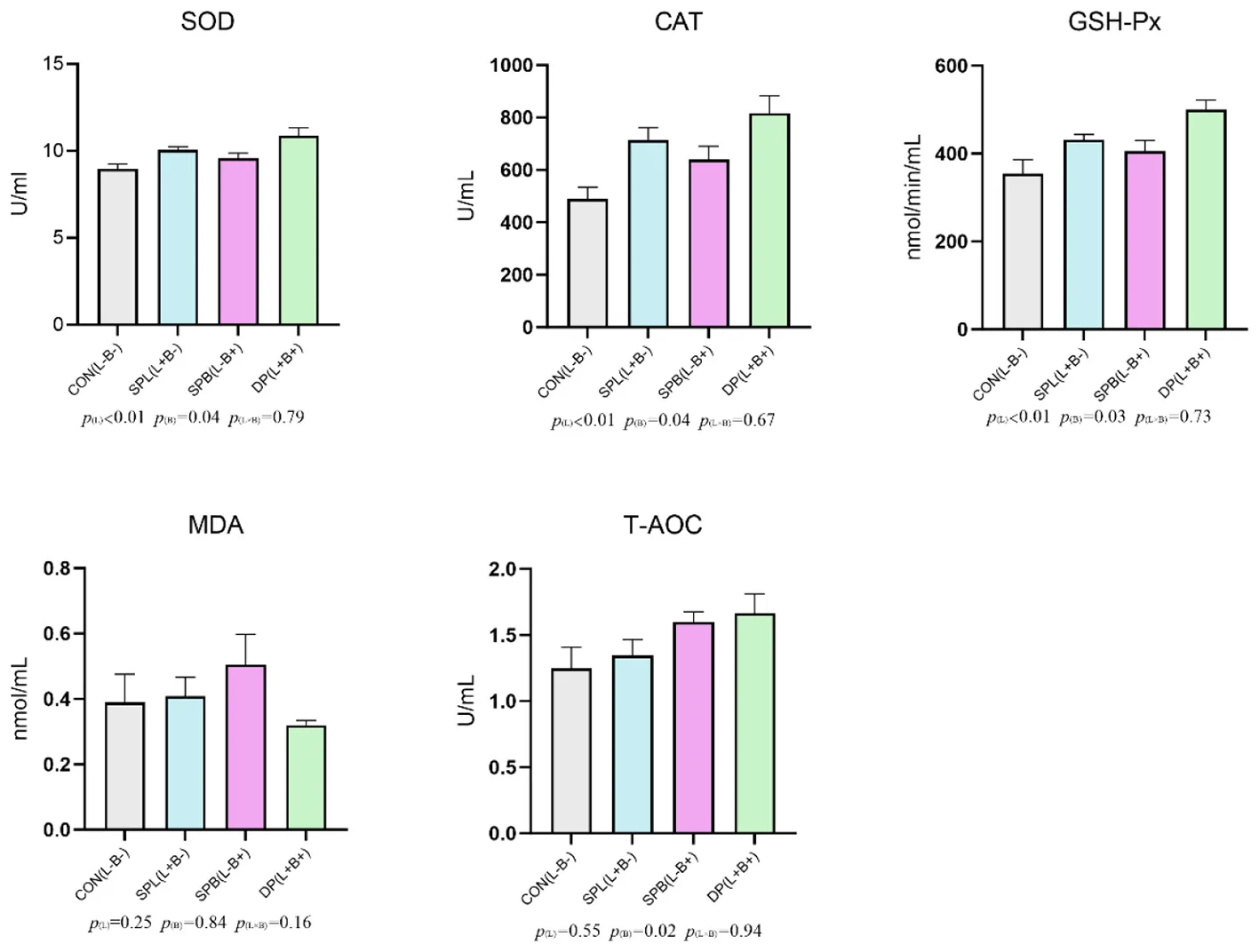
Effects of dietary probiotic supplementation on serum antioxidant capacity in growing pigs. SOD, superoxide dismutase; CAT, catalase; GSH-Px, glutathione peroxidase; MDA, malondialdehyde; T-AOC, total antioxidant capacity. The error bar indicates SEM. *n* = 6. Data were from Supplementary Tables S2, S4.

### Immune parameters and fecal calprotectin content

As shown in Figure 3, no significant L × B interaction was observed for IgG or IgM [*p*_(L × *B*)_ = 0.43 and 0.48, respectively]. Dietary supplementation with *B. velezensis* significantly increased immunoglobulin G (IgG) level [*p*_(B)_ = 0.03], while single supplementation with *L. plantarum* had no significant effect on IgG [*p*_(L)_ = 0.11]. Supplementation with *L. plantarum* significantly elevated immunoglobulin M (IgM) level [*p*_(L)_ < 0.01], and single supplementation with *B. velezensis* showed no significant influence on IgM [*p*_(B)_ = 0.10]. For IgA, *L. plantarum* significantly increased its level [*p*_(L)_ = 0.02], while no significant effect of *B. velezensis* or L × B interaction was observed [*p*_(B)_ = 0.13, *p*_(L × *B*)_ = 0.08].

**Figure 3:**
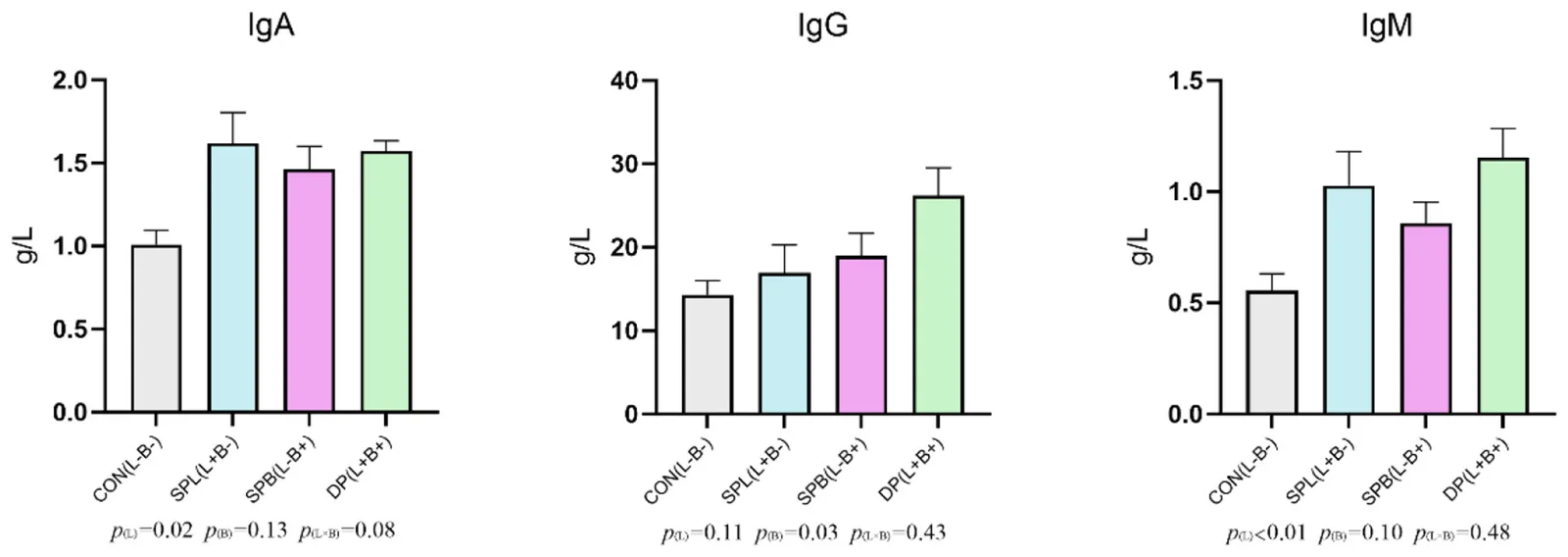
Effects of dietary probiotic supplementation on immune parameters in growing pigs. IgA, immunoglobulin A; IgG, immunoglobulin G; IgM, immunoglobulin M. The error bar indicates SEM. *n* = 6. Data were from Supplementary Table S3.

As shown in Figure 4, no significant main effects or interaction were observed for the change in fecal calprotectin (*p* > 0.05).

**Figure 4:**
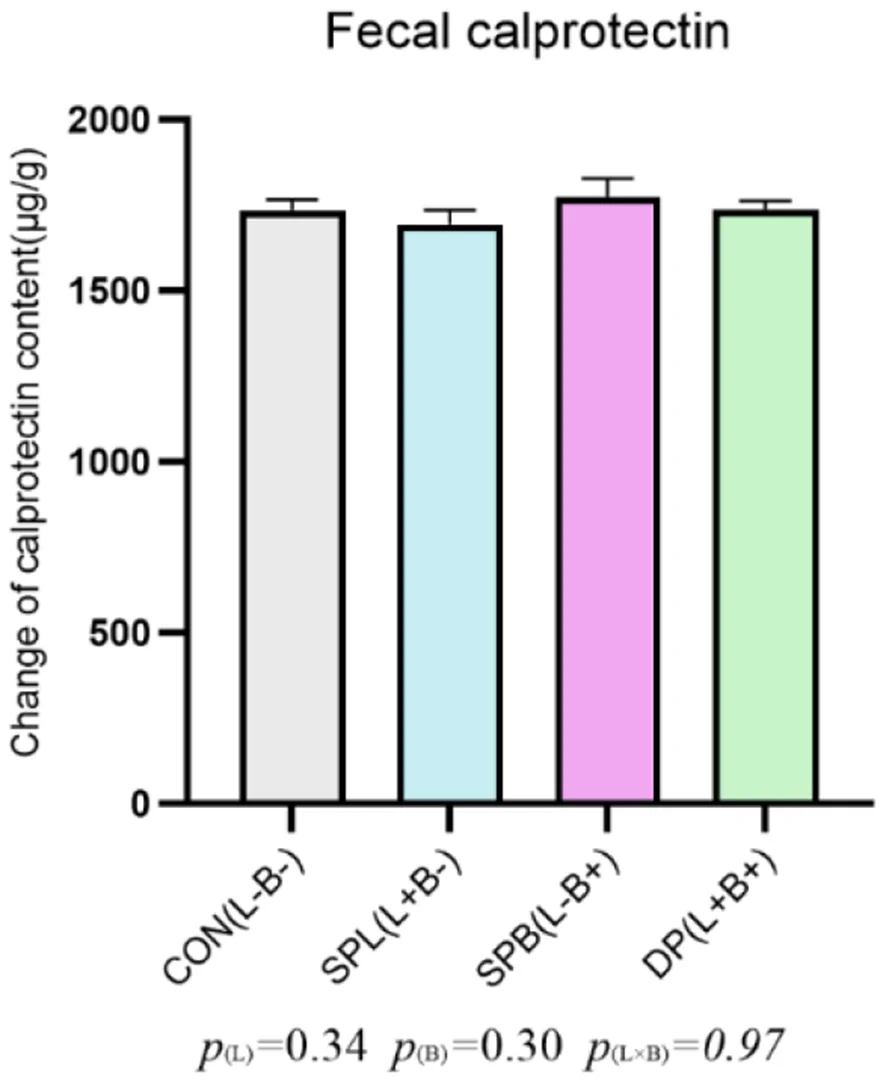
The change of calprotectin content in growing pigs supplemented diets supplemented with probiotic fermentation broth. The error bar indicates SEM. *n* = 6. Data were from Supplementary Table S4.

### Alpha and beta diversity of fecal microbiota

As shown in Figure 5, the rarefaction curves indicated that the sequencing depth was sufficient to capture the species composition and relative abundance of the intestinal microbial community.

**Figure 5:**
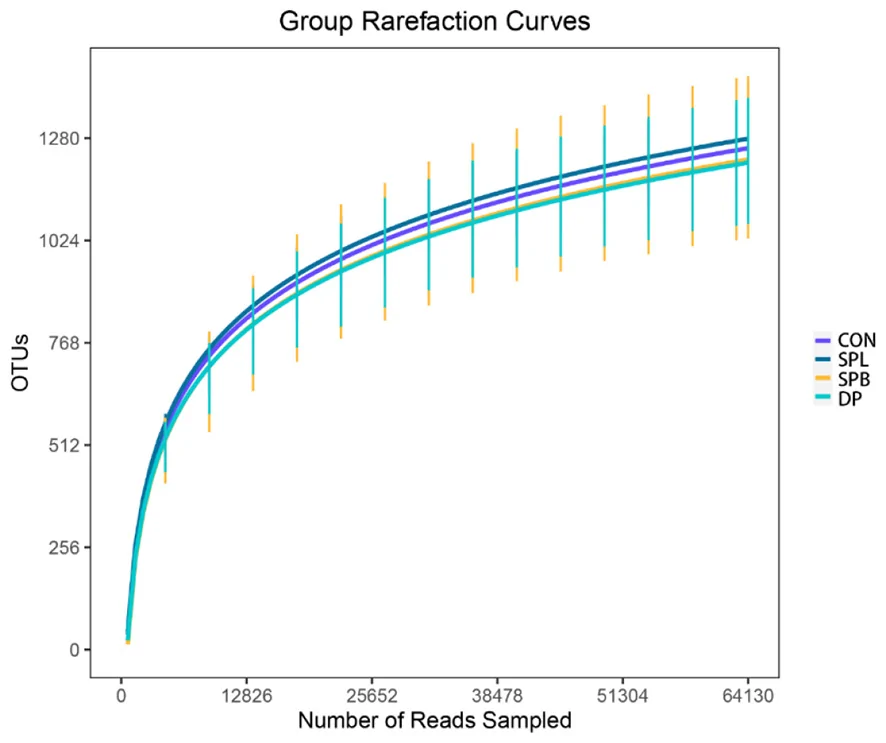
Rarefaction curves of the fecal microbial communities. *n* = 6.

As shown in Table 4, no significant L × B interaction was observed for ACE and Chao1 indices [*p*_(L × *B*)_ = 0.56 and 0.37, respectively]. Supplementation with either *L. plantarum* or *B. velezensis* significantly increased the ACE and Chao1 indices [*p*_(L)_ < 0.01, *p*_(B)_ = 0.04 and 0.03, respectively].

**Table 4:** Effects of dietary treatments on alpha diversity of fecal microbiota in growing pigs.

Items	CON(L-B-)	SPL(L+B-)	SPB(L-B+)	DP(L+B+)	*p*-value		
					L	B	L×B
ACE	714.81 ± 56.19	975.66 ± 50.95	868.87 ± 70.38	1,065.30 ± 37.27	< 0.01	0.04	0.56
Shannon	3.81 ± 0.11	4.11 ± 0.05	3.93 ± 0.12	3.88 ± 0.09	0.21	0.57	0.08
Simpson	0.93 ± 0.01	0.95 ± 0.00	0.93 ± 0.01	0.92 ± 0.01	0.57	0.10	0.10
Chao1	711.07 ± 56.78	986.64 ± 50.08	890.01 ± 69.65	1,065.19 ± 37.50	< 0.01	0.03	0.37

The results are presented as the mean ± SEM. *n* = 6.

As shown in the Venn diagram (Figure 6A), a total of 1,914 OTUs were identified across the four groups, of which 1,580 were shared among all groups. In total, 67 OTUs were unique to individual groups (CON: 24; SPL: 10; SPB: 13; DP: 20). The total number of OTUs was similar across groups, with the DP group having the highest count (1,787) and the SPL group the lowest (1,756), indicating a high degree of overlap in microbial composition among groups. Beta diversity was assessed by non-metric multidimensional scaling (NMDS) based on Bray–Curtis distance (Figure 6B). The NMDS analysis yielded a stress value of 0.1107 (< 0.2), indicating that the ordination was reliable. Although the NMDS plot showed considerable overlap of ellipses among groups, permutational multivariate analysis of variance (PERMANOVA) revealed that dietary treatment significantly affected beta diversity (pseudo-*F* = 2.13, *R*^2^ = 0.242, *p* = 0.003).

**Figure 6:**
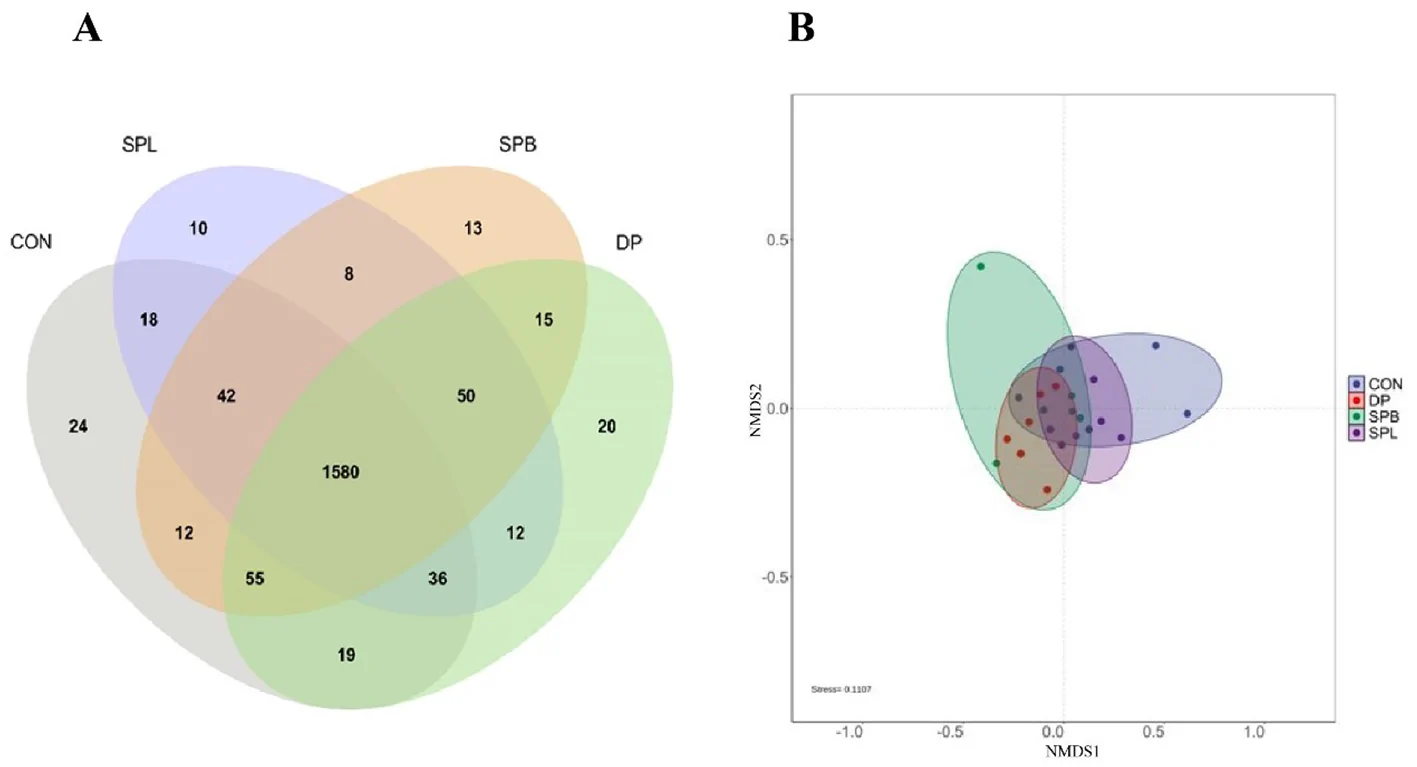
**(A)** Venn diagram of OTUs among the four groups. **(B)** NMDS analysis of fecal microbial communities based on Bray–Curtis distance. Stress 0.1107. Ellipses represent 95% confidence intervals. PERMANOVA: Pseudo-F 2.13, *R*^2^ = 0.242, *p* = 0.003. *n* = 6.

### Fecal metabolic pathway analysis

LEfSe analysis coupled with KEGG pathway functional prediction revealed distinct enrichment patterns among the treatment groups compared with the CON group (Figure 7). The SPL group exhibited significant enrichment in pathways related to fatty acid biosynthesis, ansamycin biosynthesis, branched-chain amino acid (valine, leucine, and isoleucine) biosynthesis, vancomycin group antibiotic biosynthesis, pantothenate and CoA biosynthesis, and one carbon pool by folate. No significantly enriched pathways were identified in the SPB group. In contrast, the DP group showed significant enrichment in 10 pathways, including one carbon pool by folate, homologous recombination, aminoacyl-tRNA biosynthesis, mismatch repair, peptidoglycan biosynthesis, lysine biosynthesis, D-glutamine and D-glutamate metabolism, ribosome, D-alanine metabolism, and the pentose phosphate pathway.

**Figure 7:**
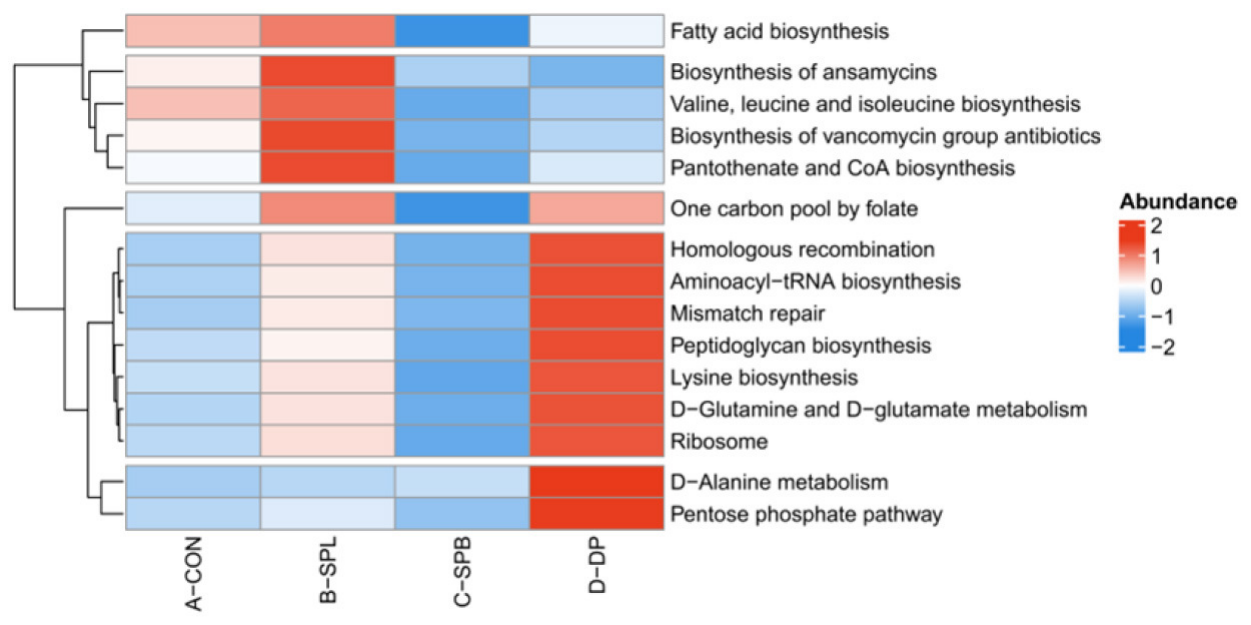
Heatmap of predicted metabolic pathway abundance based on LEfSe and KEGG analysis. Colors indicate relative enrichment levels (red, increased; blue, decreased). *n* = 6.

### Relative abundance at the phylum level

As shown in (Figure 8A), at the phylum level, *Firmicutes, Bacteroidota, Actinobacteriota* and *Spirochaetota* were the dominant phyla across all groups. Dietary supplementation with *B. velezensis* significantly increased the relative abundance of *Firmicutes, Actinobacteriota* and *Proteobacteria* [*p*_(B)_ = 0.03, 0.05 and < 0.01, respectively], and significantly reduced the relative abundance of *Bacteroidota* [*p*_(B)_ = 0.02]. Single supplementation with *L. plantarum* (L) had no significant effect on the abundances of *Firmicutes, Actinobacteriota, Proteobacteria* and *Bacteroidota* [*p*_(L)_ = 0.62, 0.60, 0.06 and 0.77, respectively]. Supplementation with *L. plantarum* significantly increased the relative abundance of *Verrucomicrobiota* [*p*_(L)_ < 0.01], while single supplementation with *B. velezensis* had no significant effect on the abundance of *Verrucomicrobiota* [*p*_(B)_ = 0.58]. *L. plantarum* significantly decreased the relative abundance of *Spirochaetota* [*p*_(L)_ = 0.02], whereas *B. velezensis* significantly increased the abundance of *Spirochaetota* [*p*_(B)_ = 0.04].

**Figure 8:**
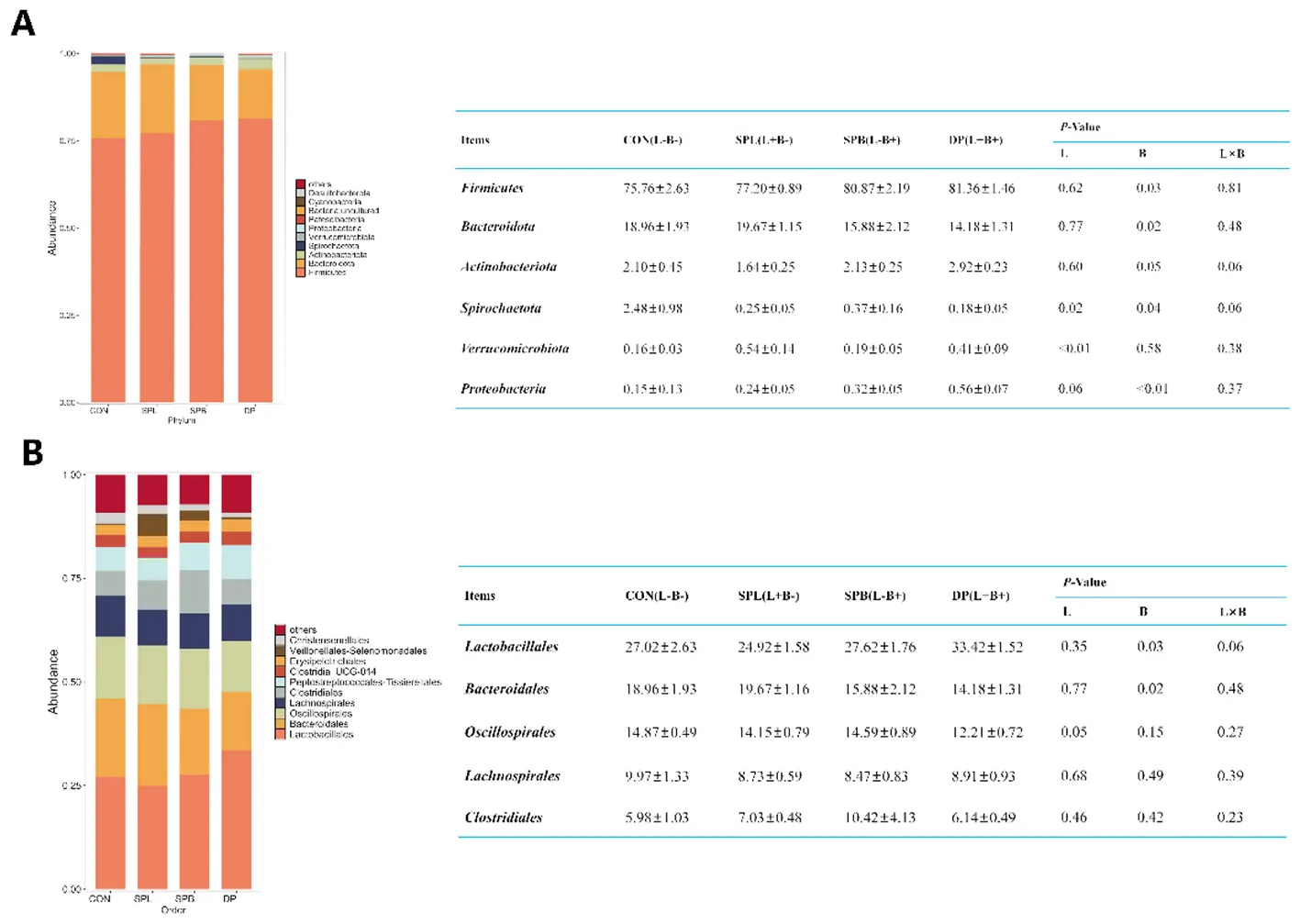
The relative abundance of OTUs in the fecal microbiota. **(A)** Richness and diversity of the fecal microbiota at the phylum level; **(B)** Richness and diversity of the fecal microbiota at the order level. *n* = 6.

At the order level (Figure 8B), *Lactobacillales, Bacteroidales, Oscillospirales, Lachnospirales*, and *Clostridiales* were the dominant orders. No significant interaction was observed for *Lactobacillales, Bacteroidales, Oscillospirales* and *Enterobacterales* at the order level [*p*_(L × *B*)_ =0.06, 0.48, 0.27 and 0.14, respectively]. Dietary supplementation with *B. velezensis* significantly increased the relative abundance of *Lactobacillales* [*P*_(B)_ = 0.03] and decreased the relative abundance of *Bacteroidales* [*p*_(B)_ = 0.02]. Single supplementation with *L. plantarum* had no significant effect on the abundances of *Lactobacillales* and *Bacteroidales* [*p*_(L)_ = 0.35 and 0.77, respectively]. Supplementation with *L. plantarum* significantly reduced the relative abundance of *Oscillospirales* [*p*_(L)_ = 0.05], while single supplementation with *B. velezensis* exerted no significant influence on the abundance of *Oscillospirales* [*p*_(B)_ = 0.15]. Meanwhile, both *L. plantarum* and *B. velezensis* significantly elevated the relative abundance of *Enterobacterales* [*p*_(L)_ < 0.01, *p*_(B)_ < 0.01].

## Discussion

Probiotics can enhance immune responses, maintain intestinal health, improve growth performance and reduce pathogen infection (Pereira et al., 2022), thereby exerting positive effects on growth performance (Kang et al., 2021; Mun et al., 2021; Song et al., 2025). Certain probiotic strains improve growth performance and feed conversion rate in pigs, and can even benefit offspring when supplemented to lactating sows (Vasquez et al., 2022; Parada et al., 2024; Chance et al., 2022). Probiotics also alleviate diarrhea severity (He et al., 2020). Multi-strain probiotics, being non-toxic, free of antimicrobial resistance, and residue-free, are recognized as safe feed additives that mitigate weaning stress, preserve intestinal integrity, and promote growth (Li et al., 2025).

In the present study, the SPB and DP groups showed significantly higher ADG and FBW than the CON group (*p* < 0.01). These findings are consistent with our previous study, which reported that multi-strain probiotics improve gut health and promote growth in growing rabbits (Ye et al., 2025). Two-way ANCOVA revealed a significant L × B interaction for ADG (*p* = 0.01). Inspection of the group means showed that the ADG in the DP group was slightly lower than that in the SPB group and comparable to that in the SPL group, consistent with the significant L × B interaction detected for ADG. However, this study still has certain limitations. Even though piglets were randomly assigned to different treatments, there were significant differences in initial body weight among groups. Initial body weight was included as a covariate in the analysis of covariance (ANCOVA) model to reduce the influence of unbalanced baseline data on growth performance indicators. However, ANCOVA cannot completely eliminate residual confounding effects. Therefore, caution is needed when interpreting results related to growth performance. Future research may adopt stratified random grouping based on initial body weight to improve the balance and comparability of baseline data across all treatment groups. Additionally, all experimental animals used in this study were gilts. Although this design controls for sex-related variation, it also limits the extrapolation of our findings to male pigs or mixed-sex populations. Future studies should include both sexes to confirm the generalizability of these results. Overall, supplementation with *Bacillus velezensis* alone or the mixture of the two strains improved growth performance compared with the control group. However, the average daily gain of the compound probiotic group was not higher than that of each single-strain group. Moreover, a significant interaction effect between *Lactobacillus plantarum* and *Bacillus velezensis* was detected. These findings suggest that the combined use of the two strains showed a non-additive and antagonistic-like tendency. A possible explanation is that the two strains may compete for similar ecological niches or nutritional resources within the gastrointestinal tract, thereby interfering with each other's efficacy. Future studies should investigate strain-specific compatibility and dose-response relationships to guide the rational design of multi-strain probiotic formulations.

Additionally, the SPL and DP groups significantly reduced diarrhea incidence (*p* < 0.05), aligning with a previous study that found that probiotics positively influence diarrhea and growth performance (Song et al., 2025). However, the reduction in diarrhea incidence was relatively modest under the experimental conditions of this study. All experimental pigs maintained good overall health, and the baseline incidence of diarrhea was low. In addition, no clinical fecal scoring or pathogen analysis was performed. Therefore, the observed reduction in diarrhea incidence alone is insufficient to serve as direct evidence of improved intestinal health. Future studies are warranted to further investigate the effects of these probiotics on intestinal health using appropriate challenge models (e.g., pathogen-induced diarrhea models) and incorporating comprehensive clinical and microbiological assessments.

Serum biochemical parameters reflect substance metabolism, organ function, and the effects of nutritional strategies. In this study, dietary supplementation with *L. plantarum* (SPL), *B. velezensis* (SPB), or their combination (DP) was evaluated for its impact on metabolism. Regarding glucose and lipid metabolism, no significant differences were observed in serum glucose, triglycerides, HDL-C, or LDL-C among groups (*p* > 0.05), indicating that probiotic supplementation did not affect basal energy metabolism. However, the SPL, SPB, and DP groups showed significantly increased serum total cholesterol and free fatty acids (*p* < 0.01), suggesting enhanced lipid mobilization and energy supply. In terms of protein metabolism, serum total protein and albumin were significantly higher in the SPB and DP groups (*p* < 0.05), and serum urea nitrogen was increased in all treatment groups (*p* < 0.01), indicating improved protein utilization and amino acid metabolism. These findings are consistent with previous reports that multi-strain probiotics increase TP and TC in poultry like ducks (Sun et al., 2022). Two-way ANOVA revealed significant L × B interactions for TC, FFA and BUN (all *p* < 0.01). For TC and FFA, the interaction was driven by a marked increase in the SPL group, whereas the DP group showed values similar to the SPB group. For BUN, both L and B exerted significant main effects, and the DP group exhibited a moderate increase relative to CON. No significant interaction was observed for TP or ALB, where only the main effect of *B. velezensis* was significant (*p* < 0.01). Overall, these results indicate that *B. velezensis* was the primary driver of improved protein metabolism, while *L. plantarum* primarily affected lipid metabolism. For BUN, the non-additive pattern contrasts with the additive patterns observed for other parameters, indicating that the metabolic effects of the two strains are pathway-dependent.

Oxidative stress is a critical challenge in modern swine production, leading to reduced growth performance, immune dysfunction, and tissue damage (Barnham et al., 2004). When reactive oxygen species exceed the body's antioxidant defense capacity, damage to biomolecules and cells can occur (Grzegorzewska et al., 2024). Probiotics exert antioxidant effects by producing antioxidant enzymes and enhancing overall antioxidant capacity (Kavyani et al., 2024). SOD and CAT scavenge superoxide anions and hydrogen peroxide, reducing oxidative stress damage (Wu et al., 2025; Choi et al., 2025), while GSH-Px maintains glutathione redox balance by inhibiting lipid peroxidation (Wei, 2024; Brandl et al., 2025). Two-way ANOVA demonstrated significant main effects of both *L. plantarum* and *B. velezensis* on serum SOD, CAT and GSH-Px activities, whereas no significant L × B interaction was detected for these parameters. These findings indicate that each probiotic strain independently contributed to enhancing antioxidant enzyme activities, without evidence of synergistic or antagonistic interaction. Although the DP group showed the highest mean values among the treatment groups, the absence of a significant interaction suggests that the combined supplementation did not produce a statistically synergistic effect beyond the additive main effects. Consistent with these findings, previous studies have reported that *L. plantarum* increases GSH-Px and CAT levels in weaned piglets (Yu et al., 2024), and that multi-strain probiotics significantly elevate SOD and GSH-Px levels (Sun et al., 2022). Overall, both probiotic strains contributed to improving antioxidant status, and the DP group exhibited the highest numerical antioxidant enzyme activities. However, because no significant L × B interaction was detected, these findings support additive rather than synergistic effects of the combined supplementation.

Immunoglobulins are key components of humoral immunity and directly reflect immune defense capacity. IgA, IgM, and IgG respectively maintain mucosal homeostasis (Lisicka et al., 2025), provide first-line humoral defense (Ji et al., 2023) and act as key inflammatory effectors (Seeling et al., 2023). In our experiment, serum IgA levels were significantly increased by *L. plantarum* supplementation [*p*_(L)_ = 0.02], with the SPL and DP groups showing higher values than the CON group. Serum IgM levels were significantly higher in the SPL and DP groups [*p*_(L)_ < 0.01]. For IgG, *B. velezensis* supplementation significantly increased its level [*p*_(B)_ = 0.03], and both SPB and DP groups tended to have higher IgG than CON. These results are similar to previous studies showing that dietary supplementation with *Bacillus* spp. and *Lactobacillus* reuteri increases serum IgA and IgG levels, thereby enhancing immune function (Kim et al., 2023; Xie et al., 2025). Collectively, dietary supplementation with *L. plantarum* (IgA and IgM), *B. velezensis* (IgG) or their combination (covering both humoral arms) promoted immune function in growing pigs.

Calprotectin is a well-established non-invasive biomarker of intestinal inflammation (Carnazzo et al., 2024; Jukic et al., 2021). Probiotic supplementation has been reported to reduce calprotectin levels and alleviate intestinal inflammation in some studies (Fallahi et al., 2013). However, no significant differences were observed in the change of fecal calprotectin among the four groups in this present study (*p* > 0.05). A plausible explanation is that all pigs remained clinically healthy throughout the trial with a relatively low baseline level of intestinal inflammation, leaving limited room for further reduction. Additionally, the duration of probiotic supplementation may not have been sufficient to induce measurable changes in calprotectin levels.

To further investigate functional changes in the intestinal microbiota, LEfSe analysis coupled with KEGG pathway prediction was performed. Compared with the CON group, the SPL group showed significant enrichment in pathways related to fatty acid biosynthesis, ansamycin biosynthesis, branched-chain amino acid biosynthesis, vancomycin group antibiotic biosynthesis, pantothenate and CoA biosynthesis, and one carbon pool by folate. These enrichment results suggest potential enhancement in lipid metabolism and energy production capacity of the gut microbiota. Nevertheless, these functional profiles are predicted from 16S rRNA gene sequencing instead of direct measurement, so cautious interpretation of these findings is necessary. The DP group showed a marked increase in both the quantity and variety of enriched pathways, which covered folate one-carbon metabolism, aminoacyl-tRNA biosynthesis, peptidoglycan biosynthesis, lysine biosynthesis, D-glutamine and D-glutamate metabolism, D-alanine metabolism, and the pentose phosphate pathway. These pathways participate in fundamental biological processes associated with DNA maintenance, protein synthesis and bacterial anabolism. However, these pathways represent core functions shared by most bacteria, so their enrichment cannot directly serve as evidence for enhanced microbial proliferation or improved host health status. Pathways involved in peptidoglycan biosynthesis and its precursor supply (D-alanine, D-glutamine/glutamate, lysine) collectively support bacterial cell wall synthesis and homeostasis (Monteiro et al., 2018; Barreteau et al., 2008). Enrichment of the pentose phosphate pathway promotes biosynthesis and reducing power generation (Loopmans et al., 2025; TeSlaa et al., 2023). The one carbon pool by folate pathway, enriched in both SPL and DP groups, plays a role in nucleic acid synthesis and cell proliferation, contributing to intestinal microbial metabolic homeostasis. No significantly enriched pathways were detected in the SPB group, suggesting that Bacillus supplementation alone had a relatively mild regulatory effect on gut microbiota function. In conclusion, compared with the intervention of a single strain, multi-strain compound probiotics could induce a broader spectrum of predicted microbial functional pathways. Nevertheless, all the above conclusions are derived from 16S rRNA gene-based functional prediction, and further validation via metagenomic, meta-transcriptomic or metabolomic analyses is still required.

The gut microbiome plays a critical role in host metabolism, immune function, and nutrient absorption (Zhang et al., 2025). Diarrhea, a common intestinal disorder closely associated with microbial imbalance, poses significant challenges to livestock production (Tian et al., 2025). In this study, alpha diversity analysis revealed that the SPL, SPB, and DP groups significantly increased ACE and Chao1 indices, indicating enrichment of low-abundance microbial taxa without altering the evenness of dominant communities. Beta diversity analysis by NMDS showed overlapping ellipses among groups. However, PERMANOVA revealed a significant effect of dietary treatment on community composition (pseudo-F = 2.13, *R*^2^ = 0.242, *p* = 0.003). At the phylum level, the DP group had significantly higher *Actinobacteriota* abundance. Although the increased abundance of these bacteria may benefit intestinal metabolic activity, current data cannot confirm that changes in intestinal function are directly caused by alterations in such bacterial communities. *Proteobacteria* abundance was increased in the SPB and DP groups [CON: 0.15%, SPL: 0.24%, SPB: 0.32%, DP: 0.56%; two-way ANOVA indicated a significant main effect of *B. velezensis*, *p*_(B)_ < 0.01]. Although these values remained within a generally acceptable range, the potential risk of *Proteobacteria* expansion should be considered. At the order level, *Lactobacillales* was enriched in the DP group, suggesting a favorable shift in lactic acid bacteria populations. *Enterobacterales* abundance was also increased in the SPB and DP groups (CON: 0.12%; SPL: 0.19%; SPB: 0.28%; DP: 0.49%), However, their absolute and relative abundances remained at low levels. Since some species belonging to *Enterobacteriales* are opportunistic pathogens, further research is required to clarify whether the observed shifts in the composition of this bacterial group carry biological significance. These changes may be influenced by dietary composition, indicating that further optimization of the probiotic formulation is needed. Although the alpha diversity analysis revealed an increase in microbial richness, the specific low-abundance taxa driving this shift have not been identified. As such, the potential functional roles of these microbes in regulating host intestinal health remain speculative. Future studies are needed to verify these effects via high-resolution microbiome profiling and functional assays. It should be noted that correlation analyses between specific microbial taxa and host physiological parameters (e.g., serum biochemical and immune indicators) were not performed in this study. The serum and fecal samples were processed independently during sample handling, making individual-level pairing unavailable. Therefore, the observed microbiota alterations and host responses are presented as parallel findings rather than causal relationships. Future studies with integrated sampling designs that preserve individual-level matching are needed to formally test microbiota–host associations.

In conclusion, dietary supplementation with probiotics, especially multi-strain compound probiotics, improves growth performance, serum biochemical parameters and antioxidant capacity, and modulates gut microbiota-related parameters in growing pigs. Probiotic treatment also remodels the gut microbiota and enhances microbial metabolic pathways. These findings provide theoretical references for developing new health-promoting feed additives in pig production. However, this study still lacks measurements of intestinal morphology, epithelial barrier integrity and histopathological changes. Current data only verify that probiotic supplementation reduces diarrhea incidence and modifies fecal microbial communities, and cannot directly confirm the improvements in intestinal structure and barrier function caused by probiotics. Follow-up research should combine intestinal histology observation, tight junction protein expression detection and intestinal permeability indicators to validate the actual effects of these probiotics on intestinal health.

## Data Availability

The 16S rRNA sequencing data generated in this study have been deposited in the NCBI Sequence Read Archive (SRA) under BioProject accession number PRJNA1498308. Other datasets used and/or analyzed during the current study are available from the corresponding author on reasonable request.
