# Supplementary material for: Dietary supplementation with Lactobacillus plantarum and Bacillus velezensis improves growth performance, antioxidant capacity, immunity and intestinal microbiota in growing-finishing pigs
Source: Front Microbiol. 2026 Sep 17;17:1913467. doi: 10.3389/fmicb.2026.1913467 (PMC13627412; doi:10.3389/fmicb.2026.1913467)
Supplement: Supplementary file 1 [file Table_1.docx]

**Table S1.** The original mean of initial body weight and final body weight

| **Items** | **CON(L-B-)** | **SPL(L+B-)** | **SPB(L-B+)** | **DP(L+B+)** | ***p*-value** |
| --- | --- | --- | --- | --- | --- |
| IBW (kg) | 26.53±0.95^a^ | 23.08±1.03^b^ | 24.52±0.77^b^ | 26.97±0.89^a^ | 0.02 |
| FBW (kg) | 65.03±0.80^ab^ | 62.99±1.17^a^ | 66.97±1.02^b^ | 68.51±0.70^c^ | <0.01 |

IBW, initial body weight; FBW, final body weight; ADG, average daily gain. The results are presented as the mean ± SEM. n = 8.

**Table S2.** Effects of dietary supplementation with probiotic fermentation broth on serum antioxidant status.

| **Items** | **CON(L-B-)** | **SPL(L+B-)** | **SPB(L-B+)** | **DP(L+B+)** | ***p*-value** | | |
| --- | --- | --- | --- | --- | --- | --- | --- |
|  |  |  |  |  | **L** | **B** | **L×B** |
| SOD (U/mL) | 8.97±0.27 | 10.07±0.18 | 9.59±0.29 | 10.86±0.45 | <0.01 | 0.04 | 0.79 |
| CAT (U/mL) | 489.62±44.63 | 714.70±47.57 | 639.20±51.36 | 817.15±66.27 | <0.01 | 0.04 | 0.67 |
| GSH-Px (nmol/min/mL) | 354.23±31.81 | 431.44±11.74 | 405.87±24.48 | 499.67±22.16 | <0.01 | 0.03 | 0.73 |
| MDA (nmol/mL) | 0.39±0.09 | 0.41±0.06 | 0.51±0.09 | 0.32±0.01 | 0.25 | 0.84 | 0.16 |
| T-AOC (U/mL) | 1.25±0.16 | 1.34±0.12 | 1.60±0.08 | 1.67±0.15 | 0.55 | 0.02 | 0.94 |

SOD: superoxide dismutase; CAT: catalase; GSH-Px: glutathione peroxidase; MDA: malondialdehyde; T-AOC: total antioxidant capacity. The results are presented as the mean ± SEM. n = 6.

**Table S3.** Effects of dietary supplementation with probiotic fermentation broth on serum immune function

| **Items** | **CON(L-B-)** | **SPL(L+B-)** | **SPB(L-B+)** | **DP(L+B+)** | ***p*-value** | | |
| --- | --- | --- | --- | --- | --- | --- | --- |
|  |  |  |  |  | **L** | **B** | **L×B** |
| IgA(g/L) | 1.01±0.09 | 1.62±0.19 | 1.47±0.14 | 1.58±0.06 | 0.02 | 0.13 | 0.08 |
| IgG(g/L) | 14.33±1.65 | 16.92±3.36 | 18.97±2.72 | 26.20±3.31 | 0.11 | 0.03 | 0.43 |
| IgM(g/L) | 0.56±0.08 | 1.03±0.15 | 0.86±0.10 | 1.15±0.13 | <0.01 | 0.10 | 0.48 |

IgA: immunoglobulin A; IgG: immunoglobulin G; IgM: immunoglobulin M. The results are presented as the mean ± SEM. n = 6.

**Table S4.** Effects of dietary supplementation with probiotic fermentation broth on fecal calprotectin content

| **Items** | **CON(L-B-)** | **SPL(L+B-)** | **SPB(L-B+)** | **DP(L+B+)** | ***p*-value** | | |
| --- | --- | --- | --- | --- | --- | --- | --- |
|  |  |  |  |  | **L** | **B** | **L×B** |
| Change in Fecal calprotectin (μg/g) | 1733.80±31.99 | 1693.67±42.60 | 1774.57±53.73 | 1737.43±24.63 | 0.34 | 0.30 | 0.97 |

The results are presented as the mean ± SEM. n = 6.
